# Hysterectomy in very obese and morbidly obese patients: a systematic review with cumulative analysis of comparative studies

**DOI:** 10.1007/s00404-015-3680-7

**Published:** 2015-03-13

**Authors:** Mathijs D. Blikkendaal, Evelyn M. Schepers, Erik W. van Zwet, Andries R. H. Twijnstra, Frank Willem Jansen

**Affiliations:** 1 Department of Gynaecology, Leiden University Medical Centre, PO Box 9600, 2300 RC Leiden, The Netherlands; 2 Department of Gynaecology, Bronovo Hospital, PO Box 96900, 2509 JH The Hague, The Netherlands; 3 Department of Medical Statistics, Leiden University Medical Centre, PO Box 9600, 2300 RC Leiden, The Netherlands

**Keywords:** Conversion, Hysterectomy, Length of hospital stay, Obesity, Postoperative complications

## Abstract

**Purpose:**

Some studies suggest that also regarding the patient with a body mass index (BMI) ≥35 kg/m^2^ the minimally invasive approach to hysterectomy is superior. However, current practice and research on the preference of gynaecologists still show that the rate of abdominal hysterectomy (AH) increases as the BMI increases. A systematic review with cumulative analysis of comparative studies was performed to evaluate the outcomes of AH, laparoscopic hysterectomy (LH) and vaginal hysterectomy (VH) in very obese and morbidly obese patients (BMI ≥35 kg/m^2^).

**Methods:**

PubMed and EMBASE were searched for records on AH, LH and VH for benign indications or (early stage) malignancy through October 2014. Included studies were graded on level of evidence. Studies with a comparative design were pooled in a cumulative analysis.

**Results:**

Two randomized controlled trials, seven prospective studies and 14 retrospective studies were included (2232 patients; 1058 AHs, 959 LHs, and 215 VHs). The cumulative analysis identified that, compared to LH, AH was associated with more wound dehiscence [risk ratio (RR) 2.58, 95 % confidence interval (CI) 1.71–3.90; *P* = 0.000], more wound infection (RR 4.36, 95 % CI 2.79–6.80; *P* = 0.000), and longer hospital admission (mean difference 2.9 days, 95 % CI 1.96–3.74; *P* = 0.000). The pooled conversion rate was 10.6 %. Compared to AH, VH was associated with similar advantages as LH.

**Conclusions:**

Compared to AH, both LH and VH are associated with fewer postoperative complications and shorter length of hospital stay. Therefore, the feasibility of LH and VH should be considered prior the abdominal approach to hysterectomy in very obese and morbidly obese patients.

## Introduction

In general, the preferred surgical approach to hysterectomy is evident [1]. In case vaginal hysterectomy (VH) is not regarded possible or in case of early-stage endometrial cancer, laparoscopic hysterectomy (LH) is associated with clear advantages over abdominal hysterectomy (AH) [1–5]. In obese patients (BMI 30.0–34.9 kg/m^2^), a similar approach to hysterectomy is considered to be best practice [6, 7]. However, no conclusive evidence exists regarding the preferred approach in the very obese and morbidly obese patients, i.e. a BMI ≥35 kg/m^2^ [8–10]. Only one of the 34 randomized controlled trials (RCT) included in the most recent Cochrane review on the surgical approach to hysterectomy, described patients with a BMI ≥35 kg/m^2^ [1, 11]. All other studies either excluded these patients from analysis or did not report the BMI.

Some non-randomized studies suggest that, compared to the AH, also this group of patients benefits most from the vaginal approach [12–15]. In daily practice, however, the VH frequently seems to be a less favourable approach due to large uterine size, (early stage) malignancy and/or expected intraoperative difficulties regarding exposure [16–18]. In more recent studies, LH was proven to be feasible and safe in these patients [2, 10, 19, 20]. Although, compared to the AH, fewer postoperative complications were found, an important point of concern is the report of a relatively high conversion rate and its suggested association with a higher postoperative morbidity [2, 8, 19, 21–24]. In contrast to these presumed better outcomes, research on the implementation and the preference of gynaecologists show that that the rate of AH increases as the BMI increases [7, 25, 26].

These dilemmas have almost become daily practice due to rising prevalence of obesity over the past decades; in Europe fluctuating between 6 and 37 % among its countries [27]. In the United States, the prevalence of BMI ≥35 kg/m^2^ remained relatively stable around 15 % [28]. Due to an increased unopposed oestrogen effect in hormonally responsive tissues, obesity can promote a number of gynaecological diseases, such as abnormal uterine bleeding and endometrial hyperplasia [29]. As a result, a higher prevalence of enlarged uteri and especially a higher incidence of endometrial carcinoma is observed among these patients [29–32]. Inherently, the number for which hysterectomy is indicated, is likely to rise over time.

Current practice shows that these controversies in literature cause diffusion in the approach to hysterectomy in these patients. To provide also the raising amount of these patients with optimal counselling and subsequent route of hysterectomy, it is necessary that conclusive evidence on this subject is obtained.

The objective of this study was to evaluate the outcomes of abdominal, laparoscopic and VH in very obese and morbidly obese patients (BMI ≥35 kg/m^2^) by means of a systematic review with cumulative analysis.

## Methods

The PubMed and EMBASE databases were systematically searched for records (last update October 9, 2014). We aimed to identify all studies on AH, LH and VH in patients with a BMI ≥35 kg/m^2^. A clinical librarian was consulted, who assisted in composing a search string including the terms (and synonyms for) body mass index, obesity, laparoscopy, abdominal, laparotomy, vaginal and hysterectomy (“Appendix 1”). No limitations regarding publication date and language were applied. All titles and subsequently the abstracts of all relevant titles were screened on relevance by two authors individually (MB and ES). Exclusion criteria during the title and abstract screening were: conference abstracts, studies without abstract, non-clinical studies (e.g. review, case report, cadaver study), a mean/median BMI <35 kg/m^2^ and studies involving extensive combined procedures (e.g. radical hysterectomy in combination with panniculectomy). Articles likely to be relevant were read in full text. Excluded were studies in which the BMI was not specified, the minimum BMI of the range was <35 kg/m^2^ (or a mean BMI <40 kg/m^2^ in case the range was not specified), multiple publications based on an overlapping cohort, studies that were not available in full text, and series of radical hysterectomies for cervical carcinoma. If the two independent reviewers did not achieve consensus on the inclusion or exclusion, a third reviewer (FWJ) was consulted.

### Study selection

From each study that was included, a predefined set of data was extracted. This consisted of study design, inclusion period (years) and indication (malignant, benign or both). In case of malignancy, it was specified if the hysterectomy was performed with or without lymph node dissection (LND). Per approach (AH, LH and VH), the number of patients and in case of LH, the type of LH [laparoscopic-assisted vaginal hysterectomy (LAVH) or total laparoscopic hysterectomy (TLH; conventional, robotic(-assisted) or both)], along with the patient and procedure characteristics, were extracted. Patient characteristics included age, BMI and uterine weight. Procedure characteristics included operating time (in minutes, skin-to-skin), blood loss (in millilitres), length of hospital stay (in days, from day of procedure), complications and conversion to laparotomy. If possible, postoperative complications were separately labelled as wound problems, dehiscence (abdominal or vaginal cuff) or wound infection. Conversion to laparotomy was defined as an intraoperative switch from a laparoscopic to an open abdominal approach. Strategic conversion (e.g. due to inadequate visibility, adhesions or additional pathology) was distinguished from reactive conversion (i.e. because of a complication) [33].

### Assessment of risk of bias

All studies were graded on the level of evidence (according to the Oxford Centre of Evidence-Based Medicine) [34]. From the highest to the lowest level, an adequately sampled (RCT) (level 1b), is followed by a low-quality RCT or observational/prospective cohort study (level 2b), an individual case–control study (3b) and a case series (and poor quality cohort or case–control study) (level 4).

### Statistical analysis

A cumulative analysis (i.e. a meta-analysis on all types of comparative studies) was conducted due to the lack of randomized evidence [35, 36]. This analysis was based on the results of all comparative studies that were included in our systematic review and was conducted using Review Manager 5.3 (Cochrane Collaboration, Copenhagen, Denmark). The pooled results of these comparative studies were expressed as risk ratios (RR) with 95 % confidence interval (CI) for dichotomous outcomes and as mean difference (MD) with 95 % CI for continuous outcomes. Regarding the latter, only results that are presented as mean with standard deviation can be included in such an analysis. Since statistical heterogeneity between the studies was expected, random effects models were used. This resulted in de most ‘conservative’ estimation of the intervention effect. Only if two or more studies could be used to estimate the effect of the pooled outcome, this outcome was reported in the Results section. The guidelines for reporting of Meta-analysis Of Observational Studies in Epidemiology (MOOSE) were followed [37].

### Hysterectomy in very obese and morbidly obese patients in our centre

All patients with a BMI ≥35.0 kg/m^2^ who underwent an elective AH, LH or VH at the Leiden University Medical Centre between January 2005 and September 2014 were also included in this study. All laparoscopic procedures were performed by two gynaecologists with extensive experience in advanced laparoscopic surgery (>200 procedures). Patients who underwent radical hysterectomy or a combined procedure (such as prolapse surgery) were excluded. All above-mentioned patient and procedure characteristics were derived by retrospective chart review. Uterine weight was derived from the pathology report. In case an actual weight was missing, the uterine volume was calculated from the pathology report or preoperative ultrasound measurements and transformed to weight by a validated model [38]. Adverse events were registered for type of complication, severity (i.e. requiring re-operation or not) and moment of onset, up to 6 weeks after discharge (i.e. marking the legitimate adverse event reporting period), according to the definitions and regulations as determined by the Guideline Adverse Events of the Dutch Society of Obstetricians and Gynaecologists [39].

The data were analysed using SPSS 20.0 statistical software (Chicago, IL, USA). A Pearson Chi square test was used to compare proportions and a student’s *T* test was used for continuous variables. To describe non-normally distributed data (kurtosis between −1 and +2) or in case Levene’s test showed no homogeneity of variance, the median and interquartile range (IQR, 25th and 75th percentiles) were used and a Mann–Whitney test was performed. A *P* < 0.05 was considered statistically significant.

## Results

**Fig. 1:**
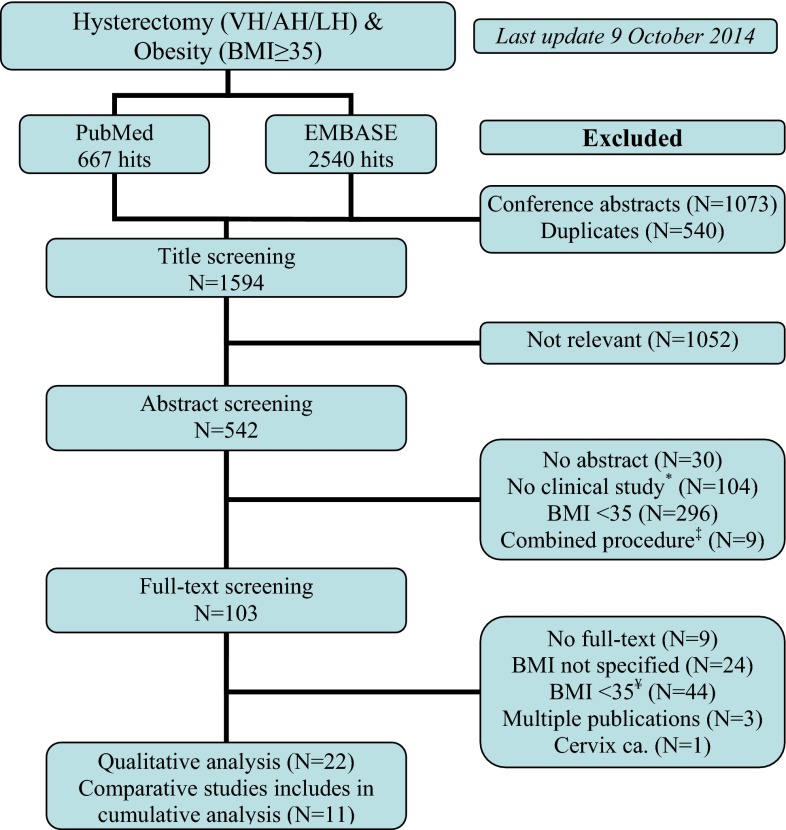
Flowchart of the search and exclusion algorithm. *Asterisk* i.e. review, case report, cadaver studies. *Double dagger* e.g. panniculectomy. *Yen sign* including mean BMI <40 kg/m^2^ if range not specified

The initial search yielded 3207 articles. After exclusion of conference abstracts (*n* = 1073), duplicates (*n* = 540), and irrelevant titles (*n* = 1052), the abstracts of 542 potentially relevant titles were screened. Based on the predefined exclusion criteria, 439 articles were excluded because no abstract was present (*n* = 30), the articles represented reviews, case reports, or cadaver studies (*n* = 104), the reported mean or median BMI of the study population was not ≥35 kg/m^2^ (*n* = 296), or the studies involved combined procedures (such as hysterectomy and panniculectomy, *n* = 9). Of the remaining 103 articles that were subjected to a full-text review, another 81 studies were excluded because the minimum BMI of the range was <35 kg/m^2^ or—in case the range was not reported—the mean BMI was <40 kg/m^2^ (*n* = 44), the BMI was not specified (*n* = 24), overlap between study populations existed (*n* = 3), no full text was available (*n* = 9), or it concerned a study on the outcomes after hysterectomy for cervical carcinoma (*n* = 1). A total of 22 articles met all inclusion criteria. Figure 1 illustrates the search and exclusion algorithm.

### Hysterectomy in very obese and morbidly obese patients in our centre

During the study period, in our centre a total of 27 AHs, 48 LHs, and five VHs were performed in patients with a BMI ≥35 kg/m^2^. In 22 % of AHs (*n* = 6) and 42 % of LHs (*n* = 20) the BMI was ≥40 kg/m^2^. Due to the low number of VHs, these procedures could not be used for further analysis.

Conversion to laparotomy was required in 12.5 % of LHs (*n* = 6). Of these, five (83 %) were for strategic considerations. The reactive conversion was performed in a patient with a BMI of 60 kg/m^2^ because of inadequate visibility during the colpotomy combined with inability to maintain the Trendelenburg position because of hypercapnia.

Patient characteristics between the groups were comparable (Table 1). Compared to AH, LH is associated with less blood loss (mean 204 ± 181 vs. 575 ± 528 mL; *P* = 0.001) and a shorter length of hospital stay (mean 3.7 ± 1.7 vs. 6.0 ± 1.8 days; *P* = 0.000). No difference in operating time was detected (mean 138 ± 38 vs. 131 ± 47 min; *P* = 0.522).

**Table 1:** Patient characteristics of all AHs and LHs performed in patients with a BMI ≥35 kg/m^2^ in our hospital from 2005 until 2014

	AH (*N* = 27)		LH (*N* = 48)		*P* value
	Mean	±SD	Mean	±SD	
Age (years)	54.8	±12.8	57.3	±11.8	0.404^a^
BMI (kg/m^2^)	37.0	36.0–39.7	38.5	36.1–44.8	0.074^b^
Uterine weight (g)	140	102–365	150	104–250	0.778^b^
Benign indication (%)	48.1 %		41.7 %		0.678^c^

*AH* abdominal hysterectomy, *LH* laparoscopic hysterectomy, *SD* standard deviation

^a^Student’s *t* test

^b^Median, interquartile range (25th and 75th percentiles) and Mann–Whitney test because of non-normal distribution

^c^Pearson Chi square

All adverse events are listed in Table 2. In 18.5 % of AHs (*n* = 5), intraoperative blood loss of >1 L was observed; all other adverse events were noted in the postoperative course. Two adverse events after LH required a re-operation (4.2 %). Compared to LH, the overall complication rate after AH was higher (40.7 vs. 16.7 %; *P* = 0.029). Among the six LHs that were converted to laparotomy, no complications were observed.

**Table 2:** Adverse events of all AHs and LHs

	AH (*N* = 27)	LH (*N* = 48)	Overall (*N* = 75)
Infection	3 (11.1 %)^a^	3 (6.3 %)^b^	6 (8.0 %)
Organ lesion	0	1 (2.1 %)^c^	1 (1.3 %)
Wound dehiscence	0	1 (2.1 %)^d^	1 (1.3 %)
Intraoperative blood loss >1 L	5 (18.5 %)	0	5 (6.7 %)
Pulmonary embolism	2 (7.4 %)	1 (2.1 %)	3 (4.0 %)
Others	1 (3.7 %)	2 (4.2 %)	3 (4.0 %)
Total	11 (40.7 %)	8 (16.7 %)	19 (25.3 %)

All adverse events did not require re-operation and occurred postoperatively, unless otherwise stated. All LHs that were converted to laparotomy were uneventful (*N* = 6)

*AH* abdominal hysterectomy, *LH* laparoscopic hysterectomy

^a^Three urinary tract infections

^b^One urinary tract infection and one aspiration pneumonia, for which antibiotics were prescribed. The third ‘infection’ regarded one single measurement of fever (39.5 °C) without focus and that normalized within 6 h without specific treatment

^c^Vesico-vaginal fistula, that needed a bladder catheter and re-operation by a urologist

^d^Readmission because of vaginal cuff dehiscence that required resuturing in the OR

### Summary of included studies

Including the data of our hysterectomies in patients with a BMI ≥5 kg/m^2^, these 23 studies resulted in a total of 2232 hysterectomies, of which 1058 were AH (14 studies), 959 LH (18 studies), and 215 VH (3 studies) [8, 14, 15, 19–22, 40–54]. Of all LHs, 952 were TLH (of which 513 were performed robotically) and 7 were LAVH. The designs of the studies were 2 RCTs, 7 prospective studies, 1 case–control study, and 13 case series or retrospective studies. In 2 studies the level of evidence was graded as 2b, in 1 study as 3b and in the remaining 20 studies as 4.

All extracted data regarding AH, LH, and VH are summarized in Tables 3, 4, 5, 6, 7 and 8, respectively (see “Appendix 2”). The pooled conversion rate was 10.6 % (95 out of 900). We calculated that 82 % of conversions (18 out of 22) could be regarded as strategic. Except for one study [52], the outcomes of all converted cases were included in the LH group (intention-to-treat analysis).

**Table 3:** Characteristics of the included studies concerning AHs (part 1 of 2)

References	Design	Level of evidence	Inclusion period	Indication	*N*	BMI	Age	OR time	Blood loss	Hospital stay	Uterus weight
Bernardini et al. [42]^a^	PS	4	2008–2010	Malign (mixed)	41	42.3 (36–66)	62 (31–86)	165 (75–295)	300 (100–3500)	4 (2–21)	NA (–)
Bijen et al. [8]^a^	RCT	2b	2007–2009	Malign (no LND)	24	NA (35–48)	NA (–)	NA (–)	NA (–)	NA (–)	NA (–)
Eisenhauer et al. [44]^a^	RS	4	1993–2006	Malign (mixed)	154	41 (35–84)	60 (25–84)	164 (40–368)	200 (40–2200)	6 (4–56)	NA (–)
Geppert et al. [19]^a^	RS	4	2005–2009	Both (no LND)	13	NA (35–51)	NA (–)	128 (65–200)	300 (100–2300)	5.7 (2–17)	NA (–)
Giugale et al. [21]^a^	RS	4	2001–2011	Both (mixed LND)	379	44±	57.8±	NA (–)	366±	NA (–)	NA (–)
Krebs et al. [47]	PS	4	1978–1982	Both (mixed LND)	21	40.3 (38–55)	52 (26–72)	195 ± 42	832.3 ± 246	12.8 ± 4	NA (–)
Obermair et al. [22]^a^	RS	4	1993–2001	Malign (mixed)	31	39.3±^b^	56.9 ± 10	127 ± 45	320 ± 240	7.9 ± 3	NA (–)
Santoso et al. [51]	PS	4	2003–2009	Malign (+LND)	88	42.7 ± 7	57.9 ± 10	117 ± 43	346 ± 319	3.5 ± 2	NA (–)
Seamon et al. [52]^a^	CC	3b	1998–2008	Malign (+LND)	162	39.9 ± 7	62 ± 12	143 ± 47	394±	3±	NA (–)
Sheth et al. [15]^a^	PS	4	1997–2007	Both (no LND)	50	45.6±	NA (–)	102±	NA (–)	5.3±	NA (–)
Showstack et al. [53]	RCT	2b	1998–2000	Benign	34	NA (35–)	NA (–)	NA (–)	NA (–)	NA (–)	NA (–)
Tinelli et al. [54]^a^	RS	4	2004–2013	Malign (+LND)	30	39 ± 8	63 ± 14	143 ± 25	125 ± 32	6.3 ± 1	NA (–)
Yu et al. [20]^a^	PS	4	2002–2003	Malign (mixed)	4	44.8±	56.5 (37–77)	142±	700±	11.5 (5–24)	NA (–)
Present study^a^	RS	4	2005–2014	Both (no LND)	27	38.2 ± 4	54.8 ± 13	131.2 ± 47	575 ± 528	6 ± 2	140 (102–365)^c^
Total					1058						

Reported values are either mean ± SD or median (min–max)

*CS* case series, *PS* prospective cohort study, *RCT* randomized controlled trial, *RS* retrospective study, *CC* case–control study, *LND* lymph node dissection

^a^Included in cumulative analysis

^b^BMI estimated based on average height of 1.70 m

^c^Interquartile range

**Table 4:** Characteristics of the included studies concerning AHs (part 2 of 2)

Author (year)	Overall complications	Intraoperative complications	Postoperative complications	Wound problem	Dehiscence	Wound infection	General remarks
	*N* (%)	*N* (%)	*N* (%)	*N* (%)	*N* (%)	*N* (%)	
Bernardini et al. [42]^a^	21 (51.2)	3 (7.3)	18 (43.9)	8 (19.5)	NA	8 (19.5)	
Bijen et al. [8]^a^	7 (29.2)	3 (12.5)	4 (16.7)	3 (12.5)	2 (8.3)	1 (4.2)	
Eisenhauer et al. [44]^a^	NA	NA	64 (41.6)	54 (35.1)	24 (15.6)	48 (31.2)	
Geppert et al. [19]^a^	NA	NA	NA	NA	NA	NA	
Giugale et al. [21]^a^	NA	NA	NA	174 (45.9)	80 (21.1)	76 (20.1)	
Krebs et al. [47]	NA	NA	17 (81.0)	5 (23.8)	4 (19.0)	4 (19.0)	Peri-umbilical incision
Obermair et al. [22]^a^	18 (58.1)	0	18 (58.1)	15 (48.4)	0	15 (48.4)	
Santoso et al. [51]	11 (12.5)	2 (2.3)	9 (10.2)	1 (1.1)	1 (1.1)	0	
Seamon et al. [52]^a^	83 (51.2)	2 (1.2)	81 (50.0)	27 (16.7)	NA	NA	
Sheth et al. [15]^a^	NA	NA	NA	9 (18.0)	4 (8.0)	NA	Article is ‘short report’
Showstack et al. [53]	NA	NA	NA	NA	NA	NA	TOSH-trial, total vs. supracervical abdominal, subgroup analysis (17 vs. 17)
Tinelli et al. [54]^a^	9 (30.0)	0	9 (30.0)	3 (10.0)	3 (10.0)	3 (10.0)	
Yu et al. [20]^a^	4 (100)	0	4 (100)	2 (50.0)	2 (50.0)	4 (100)	Article is ‘short report’
Present study^a^	11 (40.7)	5 (18.5)	6 (22.2)	0	0	0	
Total	164 (40.3)	15 (3.7)	230 (39.5)	301 (29.8)	120 (14.9)	159 (19.9)	

^a^Included in cumulative analysis

**Table 5:** Characteristics of the included studies concerning LHs (part 1 of 2)

Author (year)	Design	Level of evidence	Inclusion period	Indication	*N*	Type of LH	Techn	BMI	Age	OR time	Blood loss	Hospital stay	Uterus weight
Almeida et al. [40]	CS	4	2001–2003	Benign	7	LAVH	Conv.	45.8 (41–52)	36.9 (28–48)	109±	207 (100–350)	1.4±	141±
Almeida et al. [41]	PS	4	2011–2012	Benign	12	TLH	Robot	44.4 (40–59)	44.1 (28–67)	109.6±	146.3±	1 (1–2)	259±
Bernardini et al. [42]^a^	PS	4	2008–2010	Malign (mixed)	45	TLH	Robot	40.3 (35–75)	61 (36–87)	270 (135–470)	200 (50–1500)	2 (1–24)	NA (–)
Bijen et al. [8]^a^	RCT	2b	2007–2009	Malign (no LND)	31	TLH	Conv.	NA (35–55)	NA (–)	NA (–)	NA (–)	NA (–)	NA (–)
Eddib et al. [43]	RS	4	2010–2012	Both (mixed LND)	84	TLH	Robot	42.5±	50.4±	215.1±	79.3±	1.43±	222.7±
Eisenhauer et al. [44]^a^	RS	4	1993–2006	Malign (mixed)	25	TLH	Conv.	39 (35–49)	57 (35–79)	215 (94–330)	150 (50–500)	3 (2–7)	NA (–)
Farthing et al. [45]	RS	4	2003–2009	Malign (mixed)	45	TLH	Conv.	NA (40–)	NA (–)	75 (–)	50 (–)	2 (–)	NA (–)
Gallo et al. [46]	RS	4	2006–2010	Both (no LND)	101	TLH	Robot	44.3 (40–63)	54 (35–84)	124 (40–365)	100 (30–600)	1 (1–15)	156 (50–3543)
Geppert et al. [19]^a^	RS	4	2005–2009	Both (no LND)	23	TLH	Robot	NA (35–56)	NA (–)	136 (100–183)	50 (25–200)	1.6 (1–2)	NA (–)
Giugale et al. [21]^a^	RS	4	2001–2011	Both (mixed LND)	280	TLH	Comb.	41.7±	58.6±	NA (–)	174±	NA (–)	NA (–)
Lau et al. [48]	PS	4	2007–2009	Malign (+LND)	23	TLH	Robot	45.8 ±5	54.7 ± 10	257 ± 39	94 ± 72	2 (1–6)	204 ± 89
Nawfal et al. [49]	RS	4	2008–2010	Benign	36	TLH	Robot	NA (35–56)	NA (–)	196 (80–625)	100 (10–1000)	1 (1–5)	NA (–)
Obermair et al. [22]^a^	RS	4	1993–2001	Malign (mixed)	47	TLH	Conv.	42.1±^b^	54.6 ± 13	139 ± 51	279 ± 557	4.4 ± 3.9	NA (–)
Raiga et al. [50]	RS	4	1999	Both (no LND)	3	TLH	Conv.	49.9 (40–51)	57 (51–74)	80 (70–85)	NA (–)	3 (3–3)	NA (–)
Seamon et al. [52]^a^	CC	3b	1998–2008	Malign (+LND)	92	TLH	Robot	39.6 ± 7	58 ± 10	228 ± 43	109±	1±	NA (–)
Tinelli et al. [54]^a^	RS	4	2004–2013	Malign (+LND)	45	TLH	Conv.	38 ± 7	60 ± 11	166 ± 21	65 ± 15	3.1 ± 0.4	NA (–)
Yu et al. [20]^a^	PS	4	2002–2003	Malign (mixed)	4	TLH	Conv.	45±	58 (52–64)	153.8±	325±	4 (2–5)	NA (–)
Present study^a^	RS	4	2005–2014	Both (no LND)	48	TLH	Conv.	41 ± 6	57.3 ± 12	138 ± 38	204 ± 181	3.7 ± 1.7	150 (104–250)
Total					959								

Reported values are either mean ± SD or median (min–max)

*CS* case series, *PS* prospective cohort study, *RCT* randomized controlled trial, *RS* retrospective study, *CC* case–control study, *LND* lymph node dissection, *LAVH* laparoscopic-assisted vaginal hysterectomy, *TLH* total laparoscopic hysterectomy, *Technique* conventional, robot or combined

^a^Included in cumulative analysis

^b^BMI estimated based on average height of 1.70 m

^c^Interquartile range

**Table 6:** Characteristics of the included studies concerning LHs (part 2 of 2)

Author (year)	Overall complications	Intraoperative complications	Postoperative complications	Wound problem	Dehiscence	Wound infection	Conversion to laparotomy			
	*N* (%)	*N* (%)	*N* (%)	*N* (%)	*N* (%)	*N* (%)	*N* (%)	Reason	# SC (%)	General remarks
Almeida et al. [40]	0	NA	NA	NA	NA	NA	0		0	
Almeida et al. [41]	0	NA	NA	NA	NA	NA	1 (8.3)	Strategic	1 (100)	Conversion excluded in operating time
Bernardini et al. [42]^a^	10 (22.2)	2 (4.4)	8 (17.8)	2 (4.4)	NA	2 (4.4)	4 (8.9)	Strategic	4 (100)	
Bijen et al. [8]^a^	11 (35.5)	3 (9.7)	8 (25.8)	3 (9.7)	2 (6.5)	1 (3.2)	10 (32.3)	Unknown	NA	Performed by 16 different surgeons
Eddib et al. [43]	5 (6.0)	1 (1.2)	4 (4.8)	0	0	0	1 (1.2)	Strategic	1 (100)	
Eisenhauer et al. [44]^a^	NA	NA	3 (12.0)	3 (12.0)	0	3 (12.0)	4 (16.0)	Strategic	4 (100)	
Farthing et al. [45]	5 (9.4)	3 (5.7)	2 (3.8)	1 (1.9)	0	1 (1.9)	1 (1.9)	Strategic	1 (100)	
Gallo et al. [46]	13 (12.9)	3 (3.0)	10 (9.9)	3 (3.0)	0	3 (3.0)	1 (1.0)	Unknown	NA	
Geppert et al. [19]^a^	NA	NA	NA	NA	NA	NA	NA		NA	
Giugale et al. [21]^a^	NA	NA	NA	104 (37.1)	22 (7.9)	13 (4.6)	45 (16.1)	Unknown	NA	Converted cases: mean BMI 47.3 (vs. 40.6 non-converted). BMI > 60: conversion rate 38.5 %
Lau et al. [48]	3 (13.0)	0	3 (13.0)	2 (8.7)	0	1 (4.3)	0		0	Recovery: Hygiene regimens 3.9 days; Chores: 16.6 days, Physical activities: 18.3 days
Nawfal et al. [49]	NA	NA	NA	NA	NA	NA	NA		NA	Subgroup (total cohort 135, median BMI 30.6)
Obermair et al. [22]^a^	11 (23.4)	1 (2.1)	10 (21.3)	3 (6.4)	2 (4.3)	1 (2.1)	5 (10.6)	Both	2 (40.0)	Postoperative complications after conversion: 1 wound infection, 2 wound dehiscences, 1 atelectasis/chest infection, 1 atrial fibrillation
Raiga et al. [50]	0	0	0	0	0	0	0		0	Article in French
Seamon et al. [52]^a^	15 (16.3)	1 (1.1)	14 (15.2)	2 (2.2)	NA	NA	17 (18.5)	Unknown	NA	17 conversion excluded in further analysis; 92 Robot LHs matched to 162 laparotomies
Tinelli et al. [54]^a^	4 (8.9)	1 (2.2)	3 (6.7)	0	0	0	0		0	
Yu et al. [20]^a^	1 (25.0)	1 (25.0)	0	0	0	0	0		0	Article is ‘short report’
Present study^a^	8 (16.7)	2 (4.2)	6 (12.5)	1 (2.1)	1 (2.1)	0	6 (12.5)	Both	5 (83.3)	
Total	86 (14.5)	18 (3.1)	71 (11.8)	124 (14.1)	27 (3.6)	25 (3.2)	95 (10.6)		18 (81.8)	

*SC* strategic conversion

^a^Included in cumulative analysis

**Table 7:** Characteristics of the included studies concerning VHs (part 1 of 2)

Author (year)	Design	Level of evidence	Inclusion period	Indication	*N*	BMI	Age	OR time	Blood loss	Hospital stay	Uterus weight
Obermair et al. [22]^a^	RS	4	1993–2001	Malign (mixed)	5	NA (–)	NA (–)	NA (–)	NA (–)	NA (–)	NA (–)
Pitkin et al. [14]	RS	4	1948–1973	Benign	108	41.3 ± 5	46.2 ± 11	151 ± 41	NA (–)	NA (–)	NA (–)
Sheth et al. [15]^a^	PS	4	1997–2007	Both (no LND)	102	44±	NA (–)	80±	NA (–)	2.6±	NA (–)
Total					215						

Reported values are either mean ± SD or median (min–max)

*PS* prospective cohort study, *RS* retrospective study, *LND* lymph node dissection

^a^Included in cumulative analysis

**Table 8:** Characteristics of the included studies concerning VHs (part 2 of 2)

	Overall complications	Intra-operative complications	Postoperative complications	Wound problem	Dehiscence	Wound infection	Conversion to laparotomy			
Author (year)	*N* (%)	*N* (%)	*N* (%)	*N* (%)	*N* (%)	*N* (%)	*N* (%)	Reason	# SC (%)	General remarks
Obermair et al. [22]^a^	NA	NA	NA	NA	NA	NA	NA		NA	Excluded ‘for the aim of this analysis’
Pitkin et al. [14]	NA	NA	113.4 (105.0)	NA	NA	NA	NA		NA	BMI according to Am J Public Health (suppl), 1973
Sheth et al. [15]^a^	NA	NA	NA	0	0	NA	1 (1.0)	Reactive	0	Article is ‘short report’
Total	0	0	113.4 (105.0)	0	0	0	1 (1.0)		0 (0.0)	

*SC* strategic conversion

^a^Included in cumulative analysis

Given the fact that only 2 RCTs were found, we performed a cumulative analysis based on the included studies that were performed in a comparative design (11 out of the 22 included studies) (Tables 3, 4, 5, 6, 7 and 8, “Appendix 2”). Among these, 10 compared AH with LH, 1 compared AH with VH and none compared LH with VH.

### AH vs. LH

**Fig. 2:**
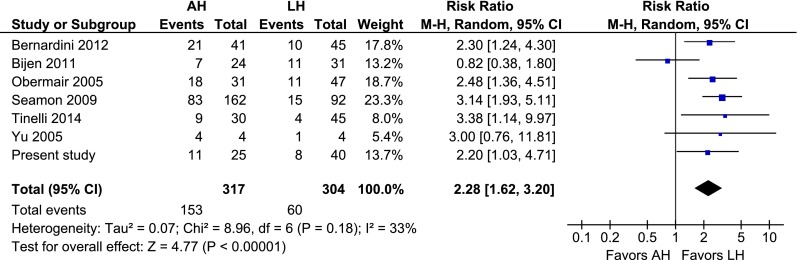
AH vs. LH, overall complication rate

**Fig. 3:**
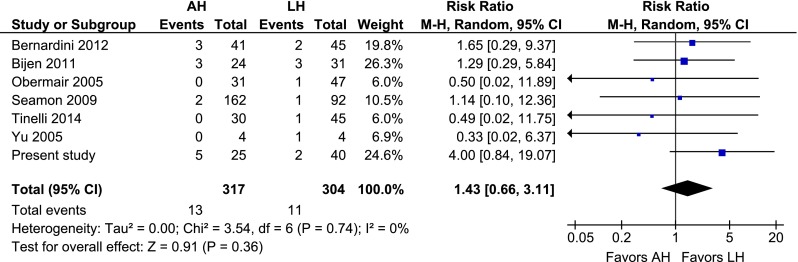
AH vs. LH, intraoperative complication rate

**Fig. 4:**
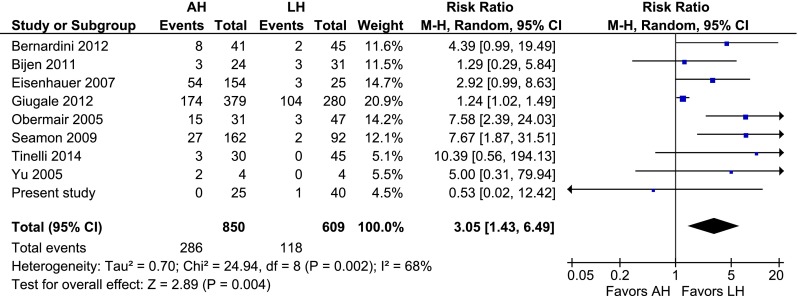
AH vs. LH, wound problem

**Fig. 5:**
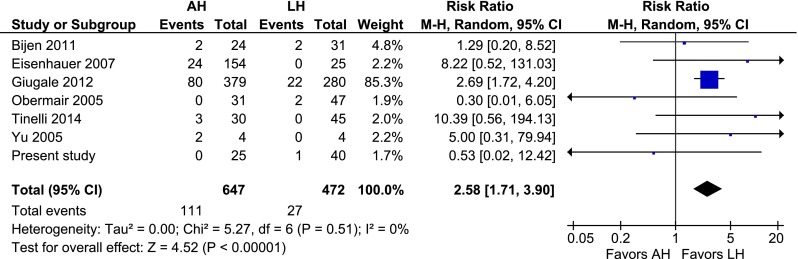
AH vs. LH, wound dehiscence (including vaginal cuff dehiscence)

**Fig. 6:**
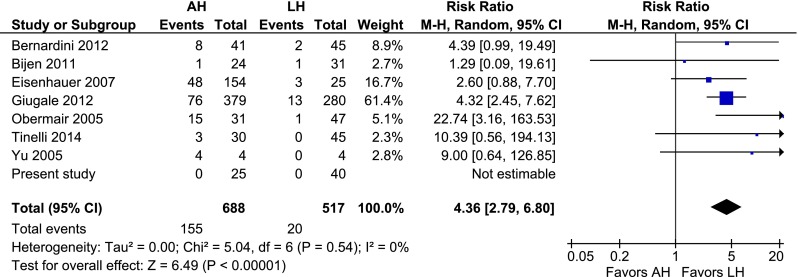
AH vs. LH, wound infection

**Fig. 7:**
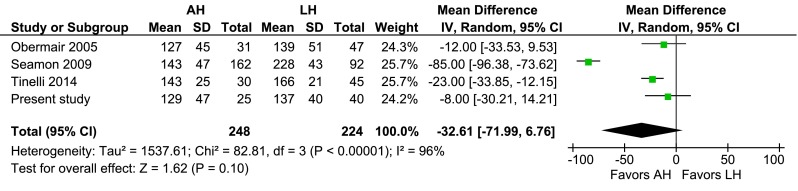
AH vs. LH, operating time

**Fig. 8:**
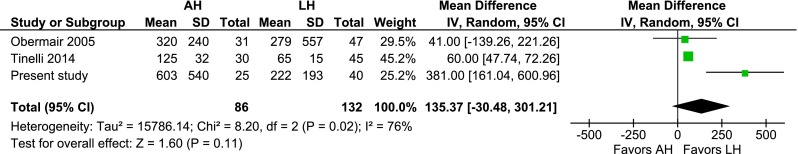
AH vs. LH, estimated blood loss

**Fig. 9:**
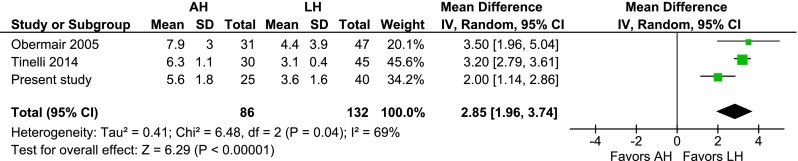
AH vs. LH, length of hospital stay

Compared to LH, AH was associated with a higher overall complication rate (RR 2.28, 95 % CI 1.62–3.20; *P* = 0.000) (Fig. 2). Intraoperative complications were rare and no difference was observed (RR 1.43, 95 % CI 0.66–3.11; *P* = 0.36) (Fig. 3). Regarding the postoperative complications, wound problems (RR 3.05, 95 % CI 1.43–6.49; *P* = 0.004), wound dehiscence (RR 2.58 95 % CI 1.71–3.90; *P* = 0.000), and wound infection (RR 4.36, 95 % CI 2.79–6.80; *P* = 0.000) all favoured LH (Figs. 4, 5, 6). No difference in operating time and estimated blood loss between AH and LH was detected (MD −33 min, 95 % CI −72–7; *P* = 0.10 and MD 135 mL, 95 % CI −30–301; *P* = 0.11, respectively) (Figs. 7, 8). The length of hospital stay was longer after AH (MD 2.9 days, 95 % CI 2.0–3.7; *P* = 0.000) (Fig. 9). No separate analysis was performed to compare benign indication and malignancy. All studies included in this cumulative analysis were for malignancy, except for one study and the hysterectomies performed in our centre [21]. Excluding the studies on robotic hysterectomies [19, 21, 42, 52] from these analyses did not cause clinically relevant differences, except for wound dehiscence (RR 2.08, 95 % CI 0.69–6.25; *P* = 0.19) and operating time (MD −19 min, 95 % CI −28 to −10; *P* = 0.000) (not shown).

### AH vs. VH

The results of one study showed more wound problems (18.0 vs. 0.0 %), more wound dehiscence (8.0 vs. 0.0 %) and a longer length of hospital stay after AH (5.3 vs. 2.6 days, Tables 3 4, 5 and 6, “Appendix 2”) [15].

## Discussion

Compared to both laparoscopic and VH, the abdominal approach in patients with a BMI ≥35 kg/m^2^ is associated with more postoperative complications and longer length of hospital stay. The majority of LHs (89 %) were completed laparoscopically. Due to better clinical outcomes, the feasibility of LH and VH should be considered prior to the abdominal approach to hysterectomy in these patients.

Although especially in patients with a BMI ≥35 kg/m^2^ a restrictive policy to abdominal surgery is warranted, the rate of AH increases as the BMI increases [7, 25, 26]. This is also reflected by the VH rates that remain stable at around 20 %, despite the fact that, in general, the vaginal approach is considered to be the preferred route to hysterectomy [1, 18]. Reasons could be a lack of experience, but also factors such as large uterine size and malignancy [55]. Since obesity is accountable for a higher incidence of both disorders, especially in the very obese and morbidly obese patients the laparoscopic approach could be the best alternative to bypass these contraindications, as confirmed by present study. Nonetheless, during laparoscopic surgery in this group of patients special considerations have to be taken into account and three-dimensional vision systems could make adequate visualisation less difficult [13, 56].

Compared to AH, both the laparoscopic and vaginal approaches are associated with a significantly lower incidence of postoperative complications. This was mainly caused by the lower risk of wound problems, such as infection and dehiscence. However, not only the incidence, but also especially the severity of these complications is a matter of concern. Unfortunately, the identified studies did not provide sufficient data to assess the severity of these complications and also other studies on this subject (mainly regarding wound infections) did show contrasting results [57–60].

Another important advantage of the laparoscopic and vaginal approach over AH is the significantly shorter length of hospital stay. Similar to the results from our centre, the cumulative analysis revealed a significant and clinically very relevant difference of approximately 3 days for the disadvantage of AH. Albeit differences in local recovery regimens and healthcare systems make comparison between studies difficult, this conclusion can be regarded valid. Firstly, it is based on differences that were found within multiple studies and secondly, they are also in line with the results of the non-comparative studies (“Appendix 2”).

Literature focusing on the outcomes of hysterectomy in patients with a BMI ≥35 kg/m^2^ proved to be scant. Instead of a meta-analysis, a cumulative analysis had to be performed on the results from prospective, non-randomized and retrospective studies [35, 36]. Since this introduced heterogeneity in our analysis, we used a random effects model to correct for the differences between studies, thereby providing the most conservative detection of differences between interventions. While these precautions have been taken into account, in our opinion, especially the major differences in complication rate and length of hospital stay cannot solely be explained by the limitations in the design of the included studies. Nonetheless, some precaution in the interpretation of our findings remains necessary. For example, the analyses on operating time, estimated blood loss and length of hospital stay are based on the results of three or four studies. Despite this, the results of these studies were similar to the outcomes of the non-comparative studies that could not be included in the cumulative analysis (“Appendix 2”).

The presumed higher conversion rate is most likely the main reason for the tendency to perform an AH instead of a LH in these patients. Conversion in general, and especially reactive conversion, is associated with more postoperative morbidity and a prolonged hospital stay [61–63]. Especially among very obese and morbidly obese patients, it is observed that conversion can result in high postoperative morbidity which has a significant impact on the quality of life, thereby obscuring the cost-effectiveness of LH over AH [8, 22, 64, 65]. The present cumulative analysis revealed a pooled conversion rate of 10.6 % and although no cost-effectiveness analysis could be performed, in our opinion, this percentage is quite comparable to the 6.5 % found in the only study that assessed cost-effectiveness with respect to conversion rate (versus a conversion rate of 32.3 % that was found to be not cost-effective) [8]. This hypothesis is further supported by the fact that the far majority (82 %) were strategic conversions. Although the risk for additional postoperative morbidity is thereby inherently minimized, further research is needed to draw more definite conclusions.

To determine superiority of VH over LH or vice versa with regard to postoperative complications, too little evidence was found. Most likely this is mainly due to the fact that VH is frequently (relatively) contraindicated due to either large uterine size or malignancy [55]. Additionally, LH was originally introduced as an alternative to AH in 1989, but at first was not accepted as an alternative for hysterectomy in very obese patients [66]. Although nowadays with the widespread implementation of LH potentially an adequately powered RCT could provide the answer, it is questionable if conducting such a study is still feasible from a methodological and ethical perspective.

The results of our systematic review with cumulative analysis finally provide sufficient evidence that also with regard to very obese and morbidly obese patients both the LH and VH result in better clinical outcomes, compared to the abdominal approach to hysterectomy. In contrast to VH, LH is considered standard of care in case of early-stage malignancy and it is less challenging to obtain adequate visualisation. Therefore, in current perspectives, LH should become the most frequently performed approach to hysterectomy in the patients with a BMI ≥35 kg/m^2^. Although a reasonable rate of conversion to laparotomy (10.6 %) was observed, hypothetically, increased experience and clustering of LH in high-volume centres might enable further improvement in the outcomes of this procedure in these patients.

## Appendix 1

Search string used for PubMed:

(“Body Mass Index”[Mesh] OR BMI[All Fields] OR “Obesity”[MeSH Terms] OR “obesity”[All Fields] OR “obese”[All Fields] OR “overweight”[MeSH Terms] OR “overweight”[All Fields] OR Quetelet[All Fields]) AND (“laparoscopy”[MeSH Terms] OR “laparoscopy”[All Fields] OR “laparoscopic”[All Fields] OR “robotic”[all fields] OR “robot”[all fields] OR “robot-assisted”[all fields] OR “abdomen”[MeSH Terms] OR “abdomen”[All Fields] OR “abdominal”[All Fields] OR “laparotomy”[MeSH Terms] OR “laparotomy”[All Fields] OR “laparotomic”[all fields] OR “vagina”[MeSH Terms] OR “vagina”[All Fields] OR “vaginal”[All Fields]) AND (“hysterectomy”[MeSH Terms] OR “hysterectomy”[All Fields] OR ((“uterus”[MeSH Terms] OR “uterus”[All Fields] OR “uterine”[all fields]) AND (“extirpation”[All Fields] OR “staging”[All Fields] OR “surgery”[All Fields]))).

Search string used for EMBASE:

(exp body mass/OR “body mass index”.mp. OR BMI.mp. OR exp obesity/OR “obesity”.mp. OR “obese”.mp. OR “overweight”.mp. OR “Quetelet”.mp.) AND (exp laparoscopic surgery/OR exp laparoscopy/OR laparoscop*.mp. OR robot*.mp. OR exp abdomen/OR abdom*.mp. OR exp laparotomy/OR laparotom*.mp. OR exp vagina/OR “vagina”.mp. OR “vaginal”.mp.) AND (exp hysterectomy/OR “hysterectomy”.mp. OR ((exp uterus/OR “uterus”.mp. OR “uterine”.mp.) AND (“extirpation”.mp. OR “staging”.mp. OR “surgery”.mp.))).

## Appendix 2

See Tables 3, 4, 5, 6, 7 and 8.
